# A Dynamic Task Scheduling Method for Multiple UAVs Based on Contract Net Protocol

**DOI:** 10.3390/s22124486

**Published:** 2022-06-14

**Authors:** Zhenshi Zhang, Huan Liu, Guohua Wu

**Affiliations:** 1 Undergraduate School, National University of Defense Technology, Changsha 410073, China; zhangzhenshi@nudt.edu.cn; 2 School of Traffic and Transportation Engineering, Central South University, Changsha 410075, China; guohuawu@csu.edu.cn

**Keywords:** multi-UAV, dynamic scheduling, contract net protocol, multi-objective optimization

## Abstract

Unmanned aerial vehicles are becoming promising platforms for disaster relief, such as providing emergency communication services in wireless sensor networks, delivering some living supplies, and mapping for disaster recovery. Dynamic task scheduling plays a very critical role in coping with emergent tasks. To solve the multi-UAV dynamic task scheduling, this paper constructs a multi-constraint mathematical model for multi-UAV dynamic task scheduling, involving task demands and platform capabilities. Three objectives are considered, which are to maximize the total profit of scheduled tasks, to minimize the time consumption, and to balance the number of scheduled tasks for multiple UAVs. The multi-objective problem is converted into single-objective optimization via the weighted sum method. Then, a novel dynamic task scheduling method based on a hybrid contract net protocol is proposed, including a buy-sell contract, swap contract, and replacement contract. Finally, extensive simulations are conducted under three scenarios with emergency tasks, pop-up obstacles, and platform failure to verify the superiority of the proposed method.

## 1. Introduction

**Figure 1:**
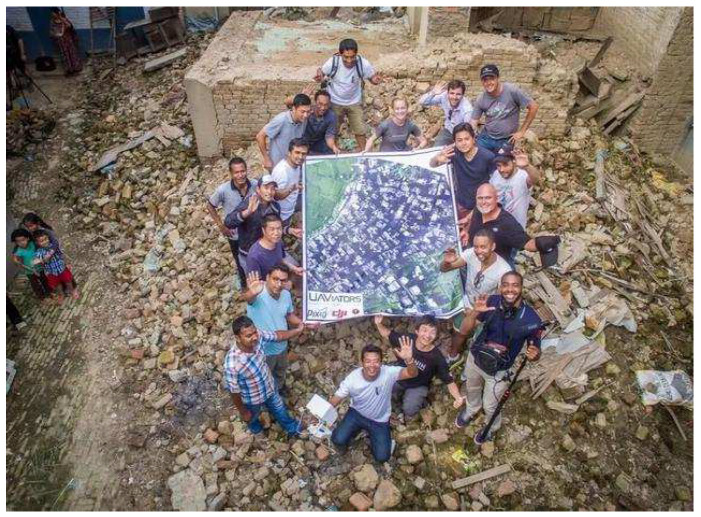
The 3D map of a destroyed village with the help of DJI drones in the 2015 Nepal earthquake.

Sudden catastrophic events, such as earthquakes, floods, terrorist attacks, etc., are always random, dynamic, destructive, and emergent. Unmanned aerial vehicles (UAVs) are flexible, low-cost, and high-efficient and can be applicable to emergency disaster relief [1,2,3,4,5,6]. In the pre-disaster stage, sensors installed on UAVs can predict the disaster’s time, geographical location, and magnitude by cooperating with current wireless sensor networks [7]. This will efficiently contribute to decreasing injury and death, material and property loss. During disasters, the base stations are usually seriously destroyed, which makes it difficult for rescuers to assist survivors efficiently. Equipped with many advanced sensors, UAVs become promising data mules for sensor network data collection, which can efficiently fly to disaster areas, hover overhand, and collect and transmit real-time information without the influence of road obstacles [8,9]. Besides, in search and rescue scenarios, intelligent sensors installed on UAVs, such as Infra-red cameras and synthetic patrol radar, can quickly discover the survivors and give the accurate positions for safer and quicker rescue. UAVs are also used to draw a detailed map of the post-disaster environment [10] in cooperation with ground devices, which facilitates disaster recovery. As shown in Figure 1, DJI drones were applied to draw a three-dimensional map of a destroyed village for disaster recovery in the 2015 Nepal earthquake.

However, there are still many challenges for multi-UAV emergency scheduling, which are as follows: (1) heterogeneous UAVs are required to complete multiple tasks, such as communication, supply delivery, mapping, and monitoring, because emergencies are often diverse and unpredicted; (2) Multiple UAVs should cooperate with each other to complete the complicated tasks; (3) Dynamic scheduling should be real-time to guarantee the successful accomplishment of tasks; (4) Energy efficiency should be taken into consideration, such as energy consumption of sensors in UAV-enabled wireless sensor networks [11,12] or fuel consumption of UAVs during the flight. Note that in this paper, we generate the short paths in two-dimensional scenarios by the APPATT [13] to reduce fuel consumption. Thus, we focus on how to reallocate tasks to multiple UAVs in real-time when confronting some emergencies.

Multi-UAV dynamic scheduling problem has been proven to be NP-hard and thus obtaining a globally optimal solution is computationally expensive [14]. Until now, many researchers around the world have put efforts into multi-UAV dynamic scheduling. Early research employed centralized methods to generate a scheme by a central server capable of gathering and spreading the whole information [15]. Nevertheless, centralized methods may place a heavy communication burden on the central server which is vulnerable to a single point of failure [16,17].

Therefore, distributed methods are often used to solve multi-UAV dynamic scheduling [18] and show excellent performance in computation and communication. Generally, among distributed algorithms, market-mechanism-based algorithms are prevailing involving auction-based algorithms and contract net protocol. Auction-based algorithms [19] are applied to generate near-optimal solutions for the task scheduling problem where one task just can be completed by one platform. However, the information of each UAV must be transmitted to the auctioneer in some way, which is time-consuming and leads to the limited network topology [20]. To cope with the issue, Choi et al. [21] proposed consensus-based decentralized auctions (CBBA) which delete the auctioneer and are distributed in each UAV, which has attracted a lot of research interest. The method includes two phases: one is the bundle-construction phase where each agent creates just a single bundle and updates it as the assignment process progresses; the other is the conflict resolution phase where agents bid on a single task and release it upon receiving a higher value in the winning bids list [22,23,24]. The two phases are in an iterative manner until the defined stopping criteria are satisfied.

Another successful market-based distributed algorithm is contract net protocol (CNP) which was proposed by Smith [25] in 1980. Agents can allocate tasks based on the market bidding mechanism (inviting tendering—bidding—winning), so that the whole multi-agent system can complete tasks with low cost and high quality [26]. Contract net protocol was employed in manufacturing systems. Sousa and Ramos [27] proposed a negotiation protocol based on CNP for job scheduling in a manufacturing system. Owliya et al. [28] presented a CNP with specific rules for job scheduling and task allocation in manufacturing applications. Vancza et al. [29] and Baker [30] applied CNP for job-shop scheduling and demonstrated its effectiveness in a natural job-shop environment.

In addition, the CNP also has been adopted in dynamic task scheduling due to its simplicity and intuitiveness. Liang et al. [31] developed an improved contract net protocol (ICNP) that incorporated multi-agent systems, which can realize dynamic task scheduling when the environment is changeable. Hong et al. [32] designed the extended contract net protocol to enhance the time efficiency and solution quality. To solve the coordinated task scheduling between manned aircrafts and UAVs, Liu et al. [33] proposed a modified contract net protocol, which can realize real-time scheduling and satisfy the requirements. In contrast, Fan et al. [34] addressed multi-UAV heterogeneous task scheduling by ICNP incorporated multi-agent systems which verified its effectiveness by extensive experiments.

Currently, many achievements have been gained in dynamic task scheduling for UAVs, which can provide theoretical and technical support for military and civil usages [35,36,37]. Nevertheless, little existing research has focused on large-scale dynamic scheduling for multiple UAVs. Hence, to solve the issue, the paper constructs a multi-constraint mathematical model for multi-UAV dynamic task scheduling, whose objectives are to maximize the total profit of scheduled tasks, to minimize the consuming time and to balance the number of scheduled tasks for multiple UAVs. A hybrid contract net protocol for dynamic task scheduling is proposed, including a buy-sell contract, swap contract, and replacement contract.

The significant contributions of this work are summarized as follows.

(1) We construct a multi-objective optimization model for multi-UAV dynamic scheduling. Three objectives are to maximize the total profit of scheduled tasks, to minimize the time consumption and to balance the number of scheduled tasks for multiple UAVs.
(2) We propose a hybrid contract net protocol involving a buy-sell contract, swap contract, and replacement contract to solve dynamic scheduling when encountering various emergencies.
(3) We conduct numerous simulations under three typical scenarios with emergency tasks, pop-up obstacles, and platform failure, to verify the effectiveness of the proposed algorithm. The simulation results demonstrate that the proposed algorithm can respond to dynamic elements and generate feasible re-scheduling schemes within milliseconds for all designed instances.

This paper is organized as follows. Section 2 constructs the mathematical formulation for multi-UAV dynamic scheduling. Section 3 proposes the hybrid contract net protocol method for multi-UAV dynamic scheduling. The effectiveness of the proposed method is then demonstrated through several simulated scenarios in Section 4. Finally, Section 5 concludes this paper.

## 2. Multi-UAV Dynamic Scheduling Model

To clearly state the multi-UAV task scheduling problem, several assumptions are made as follows.

(1) Tasks with high rewards will be scheduled firstly when the number of UAVs is limited.
(2) Bidders can evaluate their capabilities to complete tasks.
(3) Tenderees and bidders are all honest and can transmit accurate information to each other.
(4) The bid cannot be changed and canceled during the negotiation process.
(5) The communication is reliable and cannot fail, such as information loss during the negotiation process.
(6) We do not consider path re-planning when pop-up obstacles exist and the speed of UAVs is constant during the flight.

The notations used in the description of the multi-UAV task scheduling problem are summarized in Table 1. Denote *V*, *Task* and *Barrier* as the set of UAVs, tasks, and obstacles, respectively. *reward_i_* is the predefined reward of task *i*. Then, $ErstTime_{i}$ and $LastTime_{i}$ are the allowable earliest start time and latest end time of task *i*, respectively. $t_{ij}$ is the travel time from the task *i* to the task *j* and $s_{i}$ is the service time of the task *i*. The platform constraints involved in the model are flying range, energy capacity and memory capacity, which are denoted as $MaxL_{h}$, $MaxE_{h}$ and $MaxN_{h}$ (*h*: UAV index), respectively.

One decision variable denotes whether the UAV *h* flies from the task *i* to the task *j*, which is defined by a binary variable $x_{i,j}^{h}$. If the UAV *h* flies from the task *i* to the task *j*, we have $x_{i,j}^{h}=1$; otherwise, $x_{i,j}^{h}=0$. The multi-UAV task scheduling model can be formulated as follows.

### 2.1. Constraints

(1) Constraint 1: each task can only be completed at most one time.
  (1) $$\sum_{i=1}^{N_{M}+1}\sum_{h=1}^{N_{V}}x_{i,j}^{h}\leq 1(\forall j\in [1,N_{M}]\;\mathrm{and}\;j\in Z,i\neq j).$$
(2) Constraint 2: each UAV must fly to the next task after completing the current task, except for the final task.
  (2) $$\sum_{j=1}^{N_{M}}\sum_{h=1}^{N_{V}}x_{i,j}^{h}\leq 1(\forall i\in [1,N_{M}]\;\mathrm{and}\;j\in Z,i\neq j).$$
(3) Constraint 3: the order of two tasks is unique.
  (3) $$\sum_{h=1}^{N_{V}}(x_{i,j}^{h}+x_{j,i}^{h})\leq 1,\forall i,j\in [1,N_{M}]\;\mathrm{and}\;i\neq j.$$
(4) Constraint 4: $tstart_{j}$ is the start time of task *j*. $tleave_{j}$ is the departure time of task *j*. $twait_{j}$ is the waiting time of task *j*. If the former task of task *j* is task *i*, time window constraint of task *j* is as follows.
  (4) $$\left\{\begin{array}{c} tstart_{j}=tleave_{i}+t_{ij}+twait_{j}\\ tleave_{j}=tstart_{j}+s_{j}\\ tstart_{j}\geq ErstTime_{j}\\ tleave_{j}\geq LastTime_{j} \end{array}\right.$$
(5) Constraint 5: $L(project_{h})$ is the flight distance of UAV *V_h_* to accomplish tasks.
  (5) $$L(project_{h})\leq MaxL_{h}(h=1,2,\cdots ,N_{V}).$$
(6) Constraint 6: $Num\_Tasks(project_{h})$ is the number of scheduled tasks for UAV *V_h_*.
  (6) $$Num\_Tasks(project_{h})\leq MaxN_{h}(h=1,2,\cdots ,N_{V}).$$
(7) Constraint 7: $Duration(project_{h})$ is the duration time of scheduled tasks for UAV *V_h_*.
  (7) $$Duration(project_{h})\leq MaxE_{h}(h=1,2,\cdots ,N_{V}).$$

### 2.2. Objectives

During the auction progress, both the tenderee UAVs and the bidder UAVs need to calculate the performance of completing a task. Contrary to offline task scheduling, time consumption should be considered except for the total reward of scheduled tasks and the number of scheduled tasks for each UAV. Specifically, time consumption is the required time for task scheduling. Highly urgent tasks should be assigned to the appropriate UAVs in a short time. Once highly urgent tasks cannot be allocated in a reasonable time, the entire task scheduling would be affected. High profit means that when meeting the task demand, each UAV can complete many tasks with high rewards at a small cost. The number of scheduled tasks for each UAV should be balanced which can effectively avoid the rapid increase in time consumption for all UAVs. Hence, the performance of completing a task can be calculated as follows.

(8) $$U_{i}(T_{j})=\alpha *F_{i}(T_{j})+\beta *D_{i}(T_{j})+\gamma *M_{i}(T_{j}),$$

where $U_{i}(T_{j})$ is the total performance of the task $T_{j}$ for UAV $V_{i}$; $F_{i}(T_{j})$ is the profit of the task $T_{j}$; $D_{i}(T_{j})$ is the real-time performance when UAV $V_{i}$ completes the task $T_{j}$; $M_{i}(T_{j})$ is load balancing performance when UAV $V_{i}$ completes the task $T_{j}$. α,β and γ are the weight coefficients of indicators and meet the following formula, α+β+γ=1.

(1) The first objective is to maximize the net profit. The net profit can be obtained by the reward of task *i* minus the cost. The cost includes path cost (the flight distance) and risk cost (including the probability of colliding with obstacles and being discovered by hostile weapon threats). The path cost and risk cost from the task $T_{j-1}$ to task $T_{j}$ can be represented by $PathCost(V_{i},T_{j-1},T_{j})$ and $RiskCost(V_{i},T_{j-1},T_{j})$, respectively. Note that $reward(T_{j})$, $PathCost(V_{i},T_{j-1},T_{j})$ and $RiskCost(V_{i},T_{j-1},T_{j})$ can be generated after normalization. The net profit of task $T_{j}$ completed by UVA $V_{i}$ can be calculated as follows.
  (9) $$F_{i}(T_{j})=reward(T_{j})-PathCost(V_{i},T_{j-1},T_{j})-RiskCost(V_{i},T_{j-1},T_{j}).$$
(2) The second objective is to improve the real-time performance which is given below.
  (10) $$D_{i}(T_{j})=\frac{1}{e^{Time_{i}(T_{j})}},$$
  where $Time_{i}(T_{j})$ is the required time for allocating the task $T_{j}$ to the UAV $V_{i}$.
(3) During the task scheduling, it is also necessary to balance the number of scheduled tasks between UAVs. The third objective is to enhance the load balancing performance which is given below.
  (11) $$M_{i}(T_{j})=\frac{MaxN_{i}}{N_{S_{i}}},$$
  where $N_{S_{i}}$ is the number of scheduled tasks for the task scheduling scheme $S_{i}$, and $MaxN_{i}$ is the maximum number of scheduled tasks for the UAV $V_{i}$.

### 2.3. Dynamic Elements

Three dynamic elements are considered, which are emergency tasks, pop-up obstacles, and platform failure.

(1) Emergency tasks

Suppose that the set $S_{i}=\{Task_{i}^{1},Task_{i}^{2},\cdots ,Task_{i}^{n_{i}}\}$ is the offline task scheduling scheme of UAV $V_{i}$. There are $N_{t}$ emergency tasks, which can be denoted as $Etask=\{etask_{1},etask_{2},\cdots ,etask_{N_{t}}\}$. Then, the multi-UAV dynamic task scheduling can be described as: when confronting emergency tasks, task re-scheduling will be invoked requiring short time to maximize the performance of the overall system as much as possible.

(2) Pop-up obstacles

Suppose that the set $S_{i}=\{Task_{i}^{1},Task_{i}^{2},\cdots ,Task_{i}^{n_{i}}\}$ is the offline task scheduling scheme of UAV $V_{i}$. There are $N_{e}$ pop-up obstacles, which can be denoted as $Ebarrier=\{ebarrier_{1},ebarrier_{2},\cdots ,barrier_{N_{e}}\}$. Then, the multi-UAV dynamic task scheduling can be described as: when confronting pop-up obstacles, only tasks which collide with pop-up obstacles will be re-scheduled to maximize the performance of the overall system as much as possible.

(3) Platform failure

Suppose that the set $S_{i}=\{Task_{i}^{1},Task_{i}^{2},\cdots ,Task_{i}^{n_{i}}\}$ is the offline task scheduling scheme of UAV $V_{i}$. $N_{u}$ UAVs cannot work well during the implementation ($N_{u}<N_{V}$). The tasks allocated to these faulty UAVs will return to the planning center. The multi-UAV dynamic task scheduling can be described as: when some UAVs cannot work well, task re-scheduling will be invoked requiring short time to maximize the performance of the overall system as much as possible.

## 3. Hybrid Contract Net Protocol

### 3.1. Contract Net Protocol

Contract net protocol (CNP) is a management framework based on a market mechanism commonly used in the distributed control system, which was introduced into the multi-agent control field by Davis and Smith in the 1980s [25]. As a dynamic task scheduling method, CNP can find near-optimal solutions by simulating the four processes of tendering, bidding, winning the b,id and signing the contract in market economic activities [38]. When the UAV is not capable of performing some tasks, these assigned tasks are up for auction by the tenderee UAV. Then, other UAVs will bid for the tasks to be allocated. Finally, the tenderee UAV will allocate the tasks to the most suitable bidder UAVs. As a nearly global optimal scheduling method, CNP is the critical technology for UAV dynamic scheduling.

**Figure 2:**
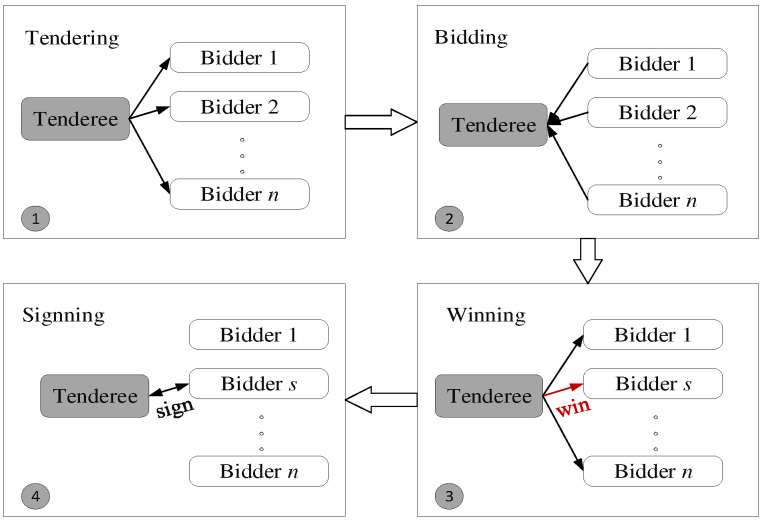
The main idea of CNP.

**Figure 3:**
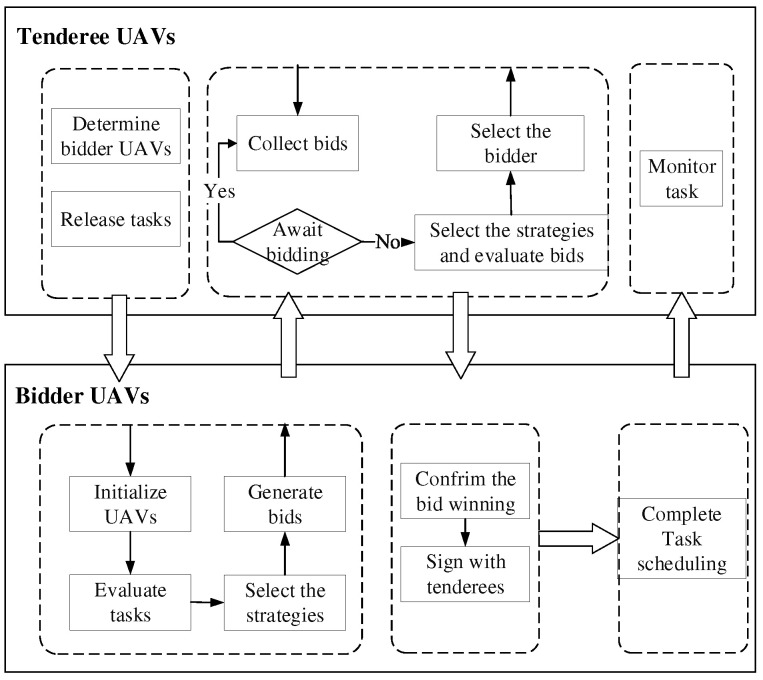
The negotiation process of CNP.

CNP is divided into tendering phase, bidding phase, winning phase and signing phase [39]. To be specific, tendering phase is that when the tenderee UAV encounters some unexpected situations during the task execution, such as pop-up obstacles, emergency tasks or platform failure, it cannot cope with the issues alone and needs assistance [40]. It will release bidding information to find suitable UAVs. The bidding phase is when other UAVs will analyze their capabilities and judge whether they can perform the tasks after receiving the bidding information. If they can deal with the dynamic issues, they will propose a reasonable bidding price based on their own performance and send it to the tenderee for bidding. The winning phase is when the tenderee will select the suitable bidder based on designed heuristics [41]. The signing phase is when the tenderee and the successful bidder reach an agreement and sign a contract to complete a task scheduling. The detailed process is described in Figure 2 and Figure 3.

### 3.2. Hybrid Contract Net Protocol

Currently, the classic buy-sell contract is generally used for multi UAV cooperative task scheduling. However, it cannot address the problem in a complex environment. To effectively solve multi-UAV coordinated task scheduling in a complex environment, this paper proposes a hybrid contract net protocol, which includes a buy-sell contract, swap contract and replacement contract.

#### 3.2.1. Buy-Sell Contract

**Figure 4:**
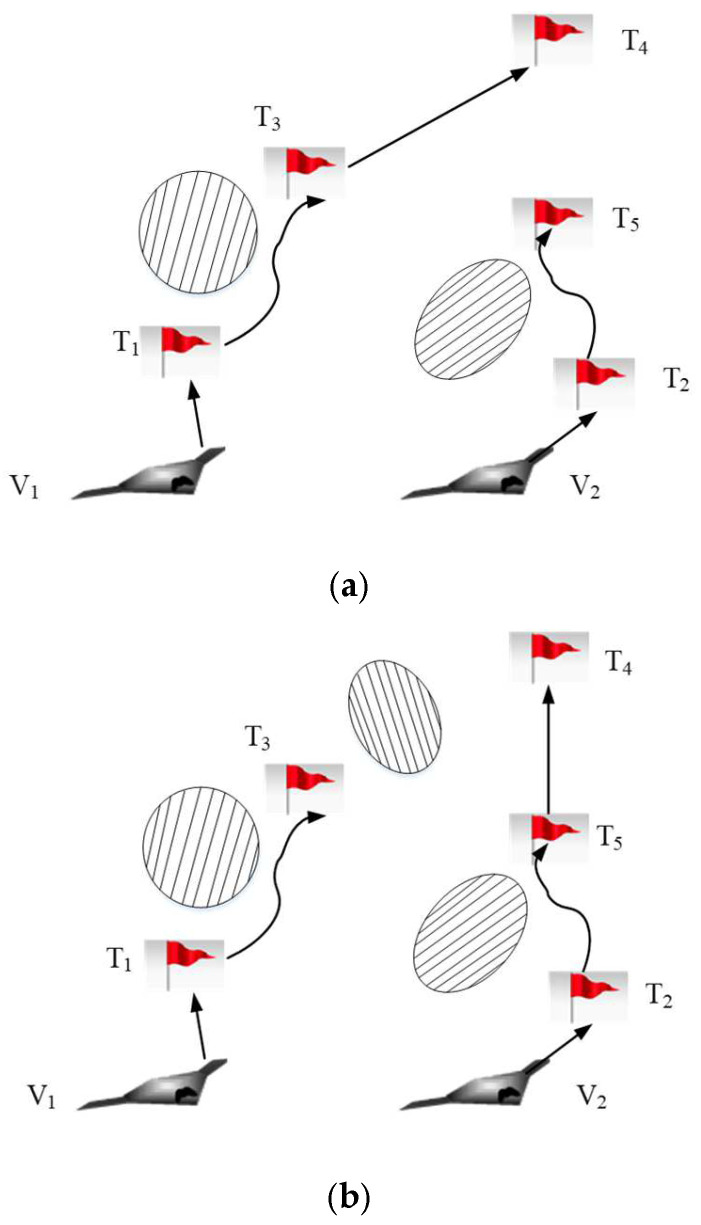
Sketch map for buy-sell contract. (**a**) Initial task scheduling scheme; (**b**) Task re-scheduling scheme.

The buy-sell contract is the primary contract type in the contract net protocol [42]. During the auction process, each UAV realizes task scheduling through the “buy-sell” way. As shown in Figure 4, the task scheduling schemes of UAV $V_{1}$ and $V_{2}$ are $\left\{T_{1},T_{3},T_{4}\right\}$ and $\left\{T_{2},T_{5}\right\}$, respectively. The UAV $V_{1}$ finds that the path from the task $T_{3}$ to the task $T_{4}$ collides with the pop-up obstacle on the way to the task $T_{3}$. The path cannot be re-planned. Thus, the UAV $V_{1}$ will release bidding information about the task $T_{4}$. We make the assumption that the UAV $V_{2}$ around the UAV $V_{1}$ is capable of performing the task $T_{4}$ based on its offline task scheduling scheme.

The buy-sell contract can be described by a quadri-tuple, $<V_{i},V_{j},T_{i,j},U_{i,j}^{sale}>$. $V_{i}$ and $V_{j}$ are a tenderee and a bidder, respectively. $T_{i,j}$ is the task that is transferred from the UAV $V_{i}$ to the UAV $V_{j}$. $U_{i,j}^{sale}$ is the performance of the whole UAV system after one auction. The procedures of the buy-sell contract are as follows.

Step 1: Initialize the performance of all UAVs (specifically, calculate the remaining capabilities and update their task scheduling schemes), and determine the only tenderee according to the maximum remaining capability.

Step 2: The tenderee $V_{i}$ will release the information about the task $T_{k}^{i}$. The low bid price of the task $T_{k}^{i}$ can be determined by the cost which the tenderee $V_{i}$ should pay for the task $T_{k}^{i}$ in the scheme $S_{i}$.

(12) $$U_{i}^{-}(T_{k}^{i})=U_{i}(S_{i})-U_{i}(S_{i}\backslash T_{k}^{i})$$

Step 3: After receiving the bidding information, the bidder $V_{j}$ will judge whether it can purchase the task $T_{k}^{i}$ according to its remaining capability. If true, the bidding price will be evaluated as follows.

(13) $$U_{j}^{+}(T_{k}^{i})=U_{j}(S_{j}\cup \{T_{k}^{i}\})-U_{j}(S_{j})$$

(14) $$U_{i,j}^{sale}(T_{k}^{i})=U_{j}^{+}(T_{k}^{i})+U_{i}^{-}(T_{k}^{i})$$

Step 4: The tenderee $V_{i}$ will evaluate the bids about the task $T_{k}^{i}$ in finite time. It will select the winner who is the highest bidder and send the information to all bidders.

(15) $$U_{i,j}^{sale}(T_{k}^{i})=\underset{q\in [1,N_{V}]}{\max}U_{i,q}^{sale}(T_{k}^{i})$$

Step 5: The winner and tenderee will sign the contract and update their task scheduling schemes.

#### 3.2.2. Swap Contract

**Figure 5:**
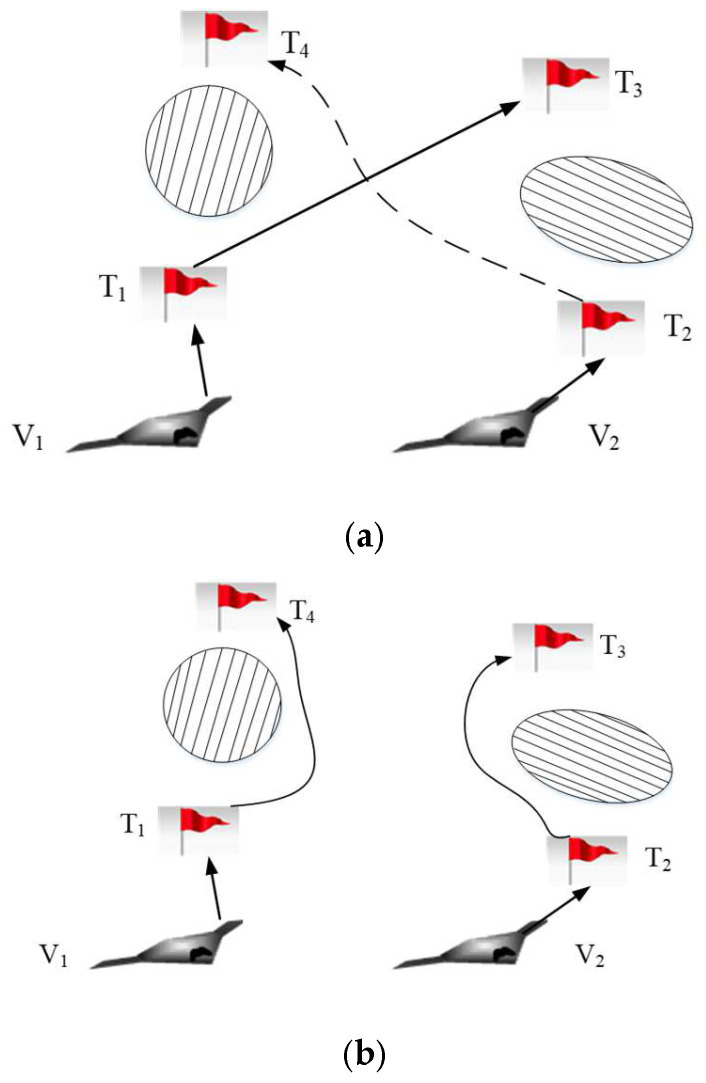
Sketch map for swap contract. (**a**) Initial task scheduling scheme; (**b**) Task re-scheduling scheme.

The buy-sell contract can be useful only when the buyers can afford the task. However, in some special cases, it is difficult to realize the task re-scheduling only by buy-sell contract. Therefore, it is necessary to adopt a swap contract to improve the efficiency of task re-scheduling [43]. As shown in Figure 5, the task scheduling schemes of UAV $V_{1}$ and $V_{2}$ are $\left\{T_{1},T_{3}\right\}$ and $\left\{T_{2}\right\}$, respectively. The maximum number of tasks which can be completed by the UAV $V_{1}$ or $V_{2}$ is 2. The emergency task $T_{4}$ is found by the UAV $V_{2}$. If the UAV $V_{2}$ performs the emergency task $T_{4}$, it will consume more fuel and can be easily detected by the enemy. At the same time, the UAV $V_{1}$ is not capable of performing the emergency task due to its limited tasks’ number. Thus, the swap contract will be applied to task re-scheduling. The task re-scheduling schemes of the UAV $V_{1}$ and $V_{2}$ are $\left\{T_{1},T_{4}\right\}$ and $\left\{T_{2},T_{3}\right\}$, respectively.

The swap contract can be described by a five-tuple, $<V_{i},V_{j},T_{i,j},T_{j,i},U_{i,j}^{swap}>$. $V_{i}$ and $V_{j}$ are a tenderee and a bidder, respectively. $T_{i,j}$ is the task which is transferred from the UAV $V_{i}$ to the UAV $V_{j}$. $T_{j,i}$ is the task which is transferred from the UAV $V_{j}$ to the UAV $V_{i}$. $U_{i,j}^{swap}$ is the performance of the whole UAV system after one auction. The procedures of the swap contract are as follows.

Step 1: Initialize the performance of all UAVs (specifically, calculate the remaining capabilities and update their task scheduling schemes), and determine the only tenderee according to the maximum remaining capability.

Step 2: The tenderee $V_{i}$ will release the information about the task $T_{k}^{i}$. The UAV $V_{j}$ will firstly use the buy-sell contract after receiving the information. If it cannot deal with the task, it will use the swap contract to swap its task $T_{l}^{j}$ with the task $T_{k}^{i}$. The performance of the UAV $V_{j}$ can be calculated by the following formula.

(16) $$U_{j}^{swap}(T_{l}^{j},T_{k}^{i})=U_{j}((S_{j}\cup \{T_{k}^{i}\})\backslash \{T_{l}^{j}\})-U_{j}(S_{j})$$

Step 3: The tenderee $V_{i}$ will evaluate its performance if it agrees to swap their tasks as follows.

(17) $$U_{i}^{swap}(T_{k}^{i},T_{l}^{j})=U_{i}((S_{i}\cup \{T_{l}^{j}\})\backslash \{T_{k}^{i}\})-U_{i}(S_{i})$$

and it will also evaluate the performance of all UAVs as follows.

(18) $$U_{i,j}^{swap}(T_{k}^{i},T_{l}^{j})=U_{i}^{swap}(T_{k}^{i},T_{l}^{j})+U_{j}^{swap}(T_{l}^{j},T_{k}^{i})$$

Step 4: The tenderee $V_{i}$ will evaluate the bids about the task $T_{k}^{i}$ in finite time. It will select the winner who is the highest bidder and send the information to all bidders.

(19) $$U_{i,j}^{swap}(T_{k}^{i},T_{l}^{j})=\underset{q\in [1,N_{V}],k\in [1,N_{P}]}{\max}\{U_{i,q}^{swap}(T_{l}^{j},T_{k}^{i})\}$$

Step 5: The winner and tenderee will sign the contract and update their task scheduling schemes.

#### 3.2.3. Replacement Contract

**Figure 6:**
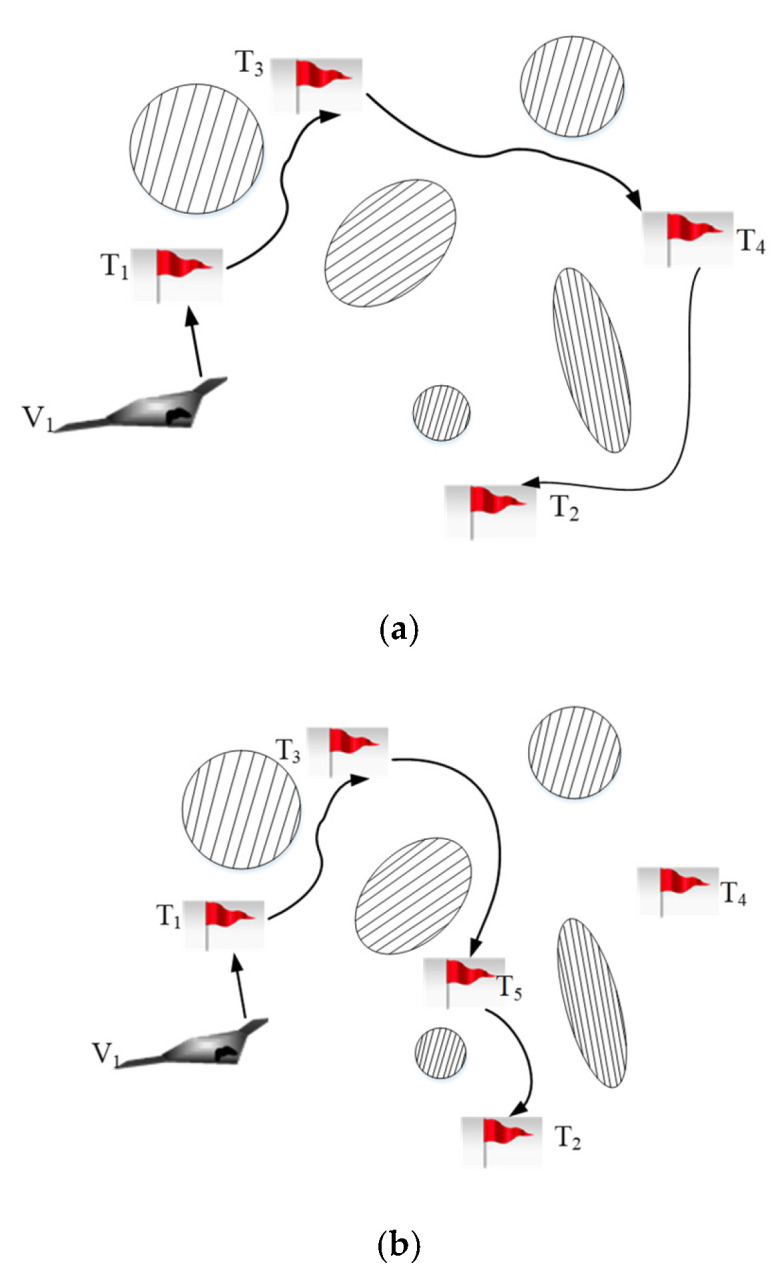
Sketch map for replacement contract. (**a**) Initial task scheduling scheme; (**b**) Task re-scheduling scheme.

If the UAV can gain a high profit after performing a new emergency task, the UAV should abandon some current tasks with low profit to bid for new tasks instead of a buy-sell contract [44]. As shown in Figure 6, the task scheduling schemes of UAV $V_{1}$ are $\left\{T_{1},T_{3},T_{4},T_{2}\right\}$. An emergency task with a high reward appears during the implementation. The replacement contract will be employed to help the UAV $V_{1}$ perform the emergency task at the cost of one task in the scheme.

The replacement contract can be described by a five-tuple, $<V_{i},V_{j},T_{i,j},T_{j,null},U_{i,j}^{replace}>$. $V_{i}$ and $V_{j}$ are a tenderee and a bidder, respectively. $T_{i,j}$ is the task which is transferred from the UAV $V_{i}$ to the UAV $V_{j}$. $T_{j,null}$ is the abandoned task from the UAV $V_{j}$. $U_{i,j}^{replace}$ is the performance of the whole UAV system after one auction. The procedures of the replacement contract are as follows.

Step 1: Initialize the performance of all UAVs (specifically, calculate the remaining capabilities and update their task scheduling schemes), and determine the only tenderee according to the maximum remaining capability.

Step 2: The tenderee $V_{i}$ will release the information about the task $T_{k}^{i}$.The UAV $V_{j}$ will use the replacement contract that it will abandon its task $T_{l}^{j}$ and perform the task $T_{k}^{i}$. The performance of the UAV $V_{j}$ can be calculated by the following formula.

(20) $$U_{j}^{replace}(T_{k}^{i},T_{l}^{j})=U_{j}((S_{j}\cup \{T_{k}^{i}\})\backslash \{T_{l}^{j}\})-U_{j}(S_{j})$$

Step 3: The tenderee $V_{i}$ will evaluate its performance if it agrees to the replacement contract as follows.

(21) $$U_{i,j}^{replace}(T_{k}^{i},T_{l}^{j})=U_{i}^{-}(T_{k}^{i})+U_{j}^{replace}(T_{k}^{i},T_{l}^{j})$$

Step 4: The tenderee $V_{i}$ will evaluate the bids about the task $T_{k}^{i}$ in finite time. It will select the winner who is the highest bidder and send the information to all bidders.

(22) $$U_{i,j}^{replace}(T_{k}^{i},T_{l}^{j})=\underset{q\in [1,N_{V}],k\in [1,N_{P}]}{\max}\{U_{i,q}^{replace}(T_{k}^{i},T_{l}^{j})\}$$

Step 5: The winner and tenderee will sign the contract and update their task scheduling schemes.

## 4. Simulation Experiments and Results

### 4.1. Emergency Tasks

(1) Task re-scheduling based on buy-sell contract

**Figure 7:**
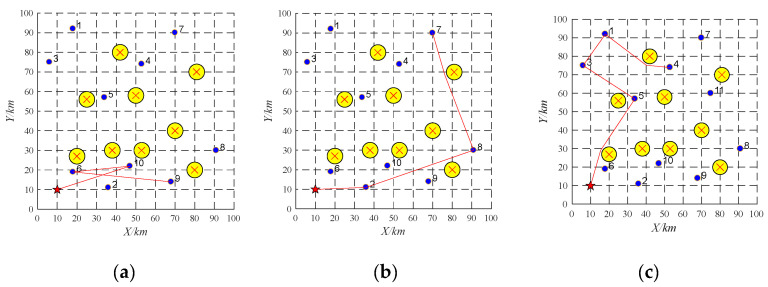
Offline task scheduling schemes. (**a**) UAV-01; (**b**) UAV-02; (**c**) UAV-03.

The proposed algorithm is coded in MATLAB and run on a PC computer with Core i5-8400 2.80 GHz CPU, 8 G memory, and Windows 10 operating system. Three UAVs are employed to perform 10 tasks. Their information is shown in Table 2 and Table 3. The offline task scheduling scheme is reported in Table 4 and Figure 7. As shown in Figure 7, the simulation scenario is applied in a 100 km × 100 km area. The yellow circles and the red star represent obstacles and a UAV base station, respectively. The purple dots represent ten tasks, and the red lines denote flight paths of each UAV. The high-quality and collision-free paths between any pair of locations can be generated by the APPATT [13].

**Figure 8:**
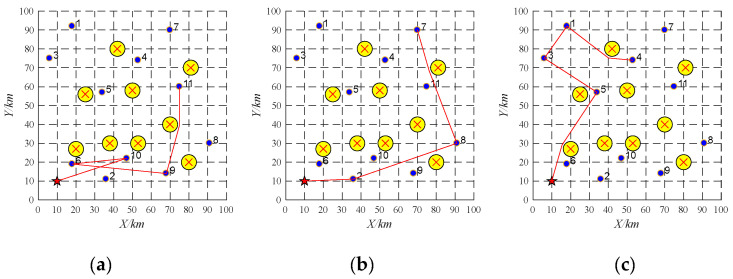
Task re-scheduling schemes with the emergency task based on buy-sell contract. (**a**) UAV-01; (**b**) UAV-02; (**c**) UAV-03.

During the implementation, the emergency task 11 (as shown in Table 5) is found and the UAV UAV-01 with the largest remaining capacity becomes the tenderee. The UAV-01 will release the information about the task 11 among the UAVs. It is found that only UAV-01 can obtain the most significant profit after it performs the emergency task. Therefore, emergency task 11 is allocated to the UAV-01. The running time of task re-scheduling is 0.003 s. The task re-scheduling schemes are as shown in Table 6 and Figure 8.

(2) Task re-scheduling based on replacement contract

**Figure 9:**
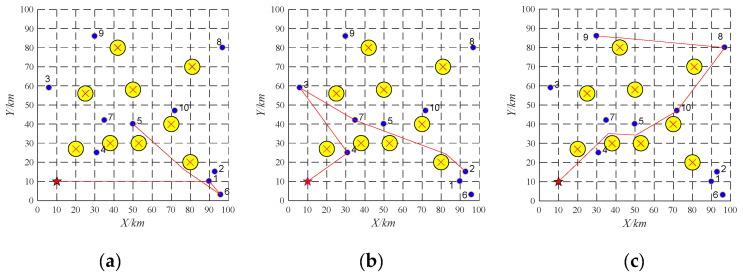
Offline task scheduling schemes. (**a**) UAV-01; (**b**) UAV-02; (**c**) UAV-03.

Three UAVs are employed to perform 10 tasks. Their information is shown in Table 2 and Table 7. The offline task scheduling scheme is reported in Table 8 and Figure 9.

**Figure 10:**
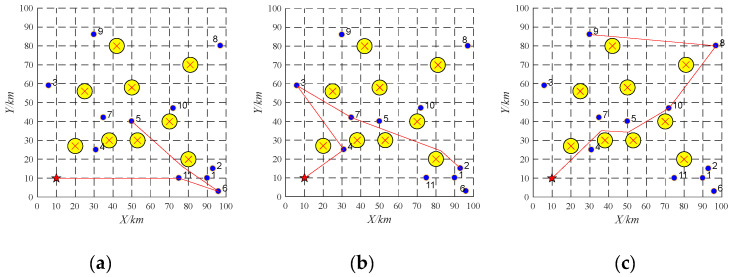
Task re-scheduling schemes with the emergency task based on replacement contract. (**a**) UAV-01; (**b**) UAV-02; (**c**) UAV-03.

During the implementation, the emergency task 11 (as shown in Table 9) is found and the UAV UAV-01 with the largest remaining capacity becomes the tenderee. The UAV-01 will release the information about the task 11 among the UAVs. It is found that no UAVs can perform the task 11 based on the buy-sell contract. Thus, the replacement contract will be employed. The task which is close to the emergency task in terms of distance and time window and whose reward is lower than the emergency task may be replaced. Through this way, the allocated task 1 is the replaced task. The running time of task re-scheduling is 0.0005 s. The task re-scheduling schemes are as shown in Table 10 and Figure 10.

### 4.2. Pop-Up Obstacles

**Figure 11:**
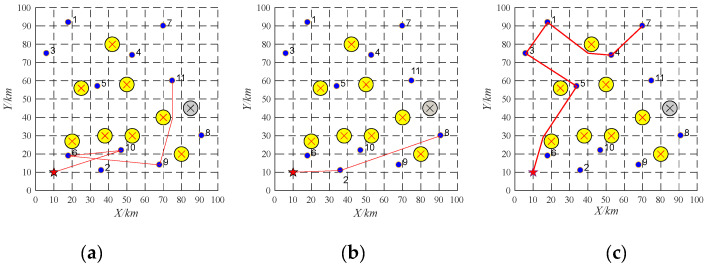
Task re-scheduling schemes with the pop-up obstacle based on replacement contract. (**a**) UAV-01; (**b**) UAV-02; (**c**) UAV-03.

In this section, the information about three UAVs and ten tasks are shown in Table 2 and Table 3. The initial task scheduling scheme of UAV-01 is 10→6→9→11. The initial task scheduling scheme of UAV-02 is 2→8→7. The initial task scheduling scheme of UAV-03 is 5→3→1→4. Task re-scheduling will be performed by buy-sell contract to deal with pop-up obstacles, whose result is reported as shown in Figure 11. During the implementation, the pop-up obstacle (the gray circle as shown in Figure 11) is found. The pop-up obstacle prohibits the UAV UAV-02 from flying from the task 8 to the task 7. Thus, the UAV UAV-02 will release the information about the task 7 and the UAV UAV-03 will win the bidding. The task re-scheduling scheme is reported as shown in Table 11.

### 4.3. Platform Failure

**Figure 12:**
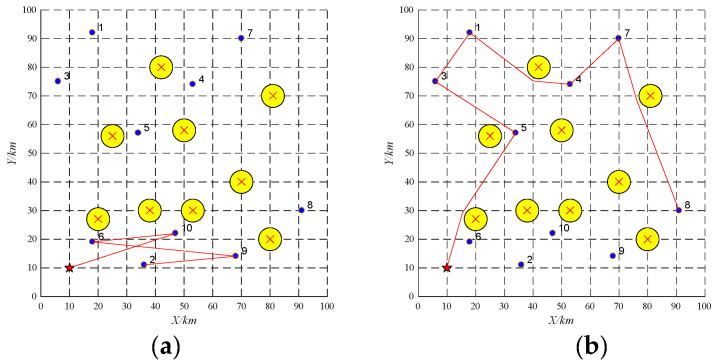
Task re-scheduling schemes with the platform failure based on hybrid contracts. (**a**) UAV-01; (**b**) UAV-03.

In this section, the initial task scheduling scheme is shown in Table 4. To be specific, the initial task scheduling scheme of UAV-01 is 10→6→9→11. The initial task scheduling scheme of UAV-02 is 2→8→7. The initial task scheduling scheme of UAV-03 is 5→3→1→4. Suppose that the UAV UAV-02 breaks down and cannot complete its assigned tasks. Its tasks 2, 8, and 7 will be sold sequentially at auction. The buy-sell contract is firstly performed. The location of task 2 is close to task 9, which means that lower travel distance will be generated if the UAV-01 accomplishes task 2 after the task 9. Besides, the time window constraint of the task 2 also can be met. Thus, the UAV UAV-01 wins the bidding and performs the task 2 after the task 9. Similarly, the tasks 7 and 8 will be allocated to the UAV UAV-03 via the buy-sell contract. The running time of task re-scheduling is 0.005 s. The final task re-scheduling scheme is shown in Table 12 and Figure 12.

As shown in Figure 12, the yellow circles and the red star represent obstacles and a UAV base, respectively. The purple dots represent ten tasks, and the red lines denote flight paths of each UAV. All the UAVs originate from one base. The UAV UAV-02 cannot depart from the base successfully and fail to complete its offline allocated tasks suddenly due to faults. Its allocated tasks will be re-allocated to the UAV-01 and UAV-03 via hybrid CNP. Figure 12a,b shows the final flight paths of each UAV which are related to their task re-scheduling schemes. Note that the flight paths may be crossed due to time windows as shown in Figure 12a.

## 5. Conclusions

To cope with the failure of the offline task scheduling scheme due to uncertain elements, a hybrid CNP is proposed to realize dynamic task scheduling. Extensive simulations are conducted under three scenarios with emergency tasks, pop-up obstacles and platform failure to verify the superiority of the proposed method. The following conclusions can be drawn from the results: (1) the hybrid CNP can respond to the dynamic elements (emergency tasks, pop-up obstacles, and platform failure) and is also capable of generating a new task scheduling scheme based on the offline scheme with about milliseconds delays for all designed cases. (2) Compared with a single contract, the hybrid CNP is more flexible and suitable for various dynamic environments, such as emergency tasks, pop-up obstacles, platform failure, and so on. In the future, more dynamic conditions should be considered, such as extreme weather, multiple platform failures, aircraft kinematics, and so on. Moreover, to get close to reality, dynamic scheduling for heterogeneous UAVs equipped with various sensors should be further discussed.

## Figures and Tables

**Table 1:** Notations.

Notations	Description
*V*	$a\;\mathrm{set}\;\mathrm{of}\;\mathrm{UAVs},\;V=\{V_{1},V_{2},\cdots ,V_{N_{V}}\}$
*h*	UAV index
*Task*	$a\;\mathrm{set}\;\mathrm{of}\;\mathrm{tasks},\;Task=\{T_{1},T_{2},\cdots ,T_{N_{M}}\}$
*i, j*	task index
*reward_i_*	the reward of task *i*
$\left[ErstTime_{i},LastTime_{i}\right]$	the allowable earliest start time and latest end time of task *i*
$s_{i}$	the service time of task *i*
$t_{ij}$	the travel time from task *i* to task *j*
$MaxL_{h},MaxE_{h},MaxN_{h}$	flying range, energy capacity and memory capacity of UAV *h*

**Table 2:** Parameter of UAVs.

UAV	Base	Flying Range (km)	Speed (km/m)	Memory Capacity (Unit)	Energy Capacity (s)
UAV-01	(10,10)	200	5	5	100
UAV-02		200	5	5	100
UAV-03		200	5	5	100

**Table 3:** Parameter of Initial Tasks.

No.	Coordinate	Service Time (s)	Time Window		Reward
			Erst Time	Last Time	
1	(18,92)	3.84	8:39:00	9:11:00	105
2	(36,11)	1.98	8:55:00	9:01:00	114
3	(6,75)	1.36	8:23:00	8:48:00	65
4	(53,74)	0.19	9:04:00	9:11:00	99
5	(34,57)	3.37	8:12:00	8:55:00	17
6	(18,19)	2.15	8:15:00	9:03:00	141
7	(70,90)	2.26	9:18:00	9:36:00	29
8	(91,30)	3.05	8:50:00	9:47:00	40
9	(68,14)	0.30	8:29:00	9:27:00	120
10	(47,22)	1.58	8:24:00	8:50:00	74

**Table 4:** Offline Task Scheduling Schemes.

UAV	Flying Range (km)	Scheduling Scheme	Reward
UAV-01	118	10→6→9	335
UAV-02	147	2→8→7	183
UAV-03	149	5→3→1→4	283

**Table 5:** Parameter of the Emergency Tasks.

No.	Coordinate	Service Time (s)	Time Window		Reward
			Erst Time	Last Time	
11	(75,60)	0.56	8:53:00	9:20:00	88

**Table 6:** Task Re-scheduling Schemes based on Buy-sell Contract to Deal with Emergency Tasks.

UAV	Flying Range (km)	Scheduling Scheme	Reward
UAV-01	165	10→6→9→11	423
UAV-02	147	2→8→7	183
UAV-03	149	5→3→1→4	283

**Table 7:** Parameter of Tasks.

No.	Coordinate	Service Time (s)	Time Window		Reward
			Erst Time	Last Time	
1	(90,10)	4.65	8:23:00	8:35:00	18
2	(93,15)	0.47	9:19:00	9:54:00	81
3	(6,59)	4.84	8:22:00	9:10:00	89
4	(31,25)	0.17	8:19:00	8:40:00	148
5	(50,40)	4.42	8:35:00	8:55:00	57
6	(96,3)	1.36	8:30:00	9:06:00	127
7	(35,42)	0.87	9:22:00	9:14:00	98
8	(97,80)	2.73	9:26:00	9:48:00	96
9	(30,86)	4.71	9:27:00	9:59:00	17
10	(72,47)	2.80	8:01:00	9:00:00	148

**Table 8:** Offline Task Scheduling Schemes.

UAV	Flying Range (km)	Scheduling Scheme	Reward
UAV-01	148	1→6→5	202
UAV-02	167	4→ 3→ 7→2	416
UAV-03	183	10→8→9	261

**Table 9:** Parameter of the Emergency Tasks.

No.	Coordinate	Service Time (s)	Time Window		Reward
			Erst Time	Last Time	
11	(75,10)	0.56	8:22:00	8:38:00	88

**Table 10:** Task Re-scheduling Schemes based on Replacement Contract to Deal with Emergency Tasks.

UAV	Flying Range (km)	Scheduling Scheme	Reward
UAV-01	126	11→6→5	272
UAV-02	167	4→3→7→2	416
UAV-03	183	10→8→9	261

**Table 11:** Task Rescheduling Schemes based on Buy-sell Contract to Deal with Pop-up Obstacles.

UAV	Flying Range (km)	Scheduling Scheme	Reward
UAV-01	165	10→6→9→11	423
UAV-02	84	2→8	154
UAV-03	183	5→3→1→4→7	206

**Table 12:** Task Re-scheduling Schemes based on Hybrid Contracts to Deal with Platform Failure.

UAV	Flying Range (km)	Scheduling Scheme	Reward
UAV-01	150	10→6→9→2	449
UAV-03	235	5→3→1→4→7→8	352

## Data Availability

Not applicable.
